# A second ortho­rhom­bic polymorph of 4-{[(1*E*,2*E*)-3-(4-meth­oxy­phen­yl)prop-2-en-1-yl­idene]amino}-1,5-dimethyl-2-phenyl-1*H*-pyrazol-3(2*H*)-one

**DOI:** 10.1107/S2056989026001039

**Published:** 2026-02-05

**Authors:** M. G. Shankar, A. Subashini, R. Kumaravel, T. C. Sabari Girisun, K. Ramamurthi, Aurélien Crochet, Helen Stoeckli-Evans

**Affiliations:** a PG and Research Department of Physics Srimad Andavan Arts and Science College (Autonomous) Affiliated to Bharathidasan University, Tiruchirappalli - 620 005 Tamil Nadu India; b Department of Physics Swami Dayananda College of Arts and Science Affiliated to Bharathidasan University, Manjakkudi - 612 610 Tamil Nadu India; c Department of Physics, Annapoorana Engineering College (Autonomous), Salem - 636308, Tamil Nadu, India; d Nanophotonics Laboratory Department of Physics Bharathidasan University, Tiruchirappalli - 620 024 Tamil Nadu India; e Crystal Growth and Thin Film Laboratory Department of Physics Bharathidasan University, Tiruchirappalli - 620 024 Tamil Nadu India; f Chemistry Department, University of Fribourg, Chemin du Musée 9, CH-1700 Fribourg, Switzerland; g Institute of Physics, University of Neuchâtel, Rue Emile-Argand 11, CH-2000 Neuchâtel, Switzerland; University of Aberdeen, United Kingdom

**Keywords:** crystal structure, 4-amino­anti­pyrine, acryl­aldehyde, polymorphism, positional disorder, hydrogen bonding, Hirshfeld surface, fingerprint plots

## Abstract

The second ortho­rhom­bic polymorph of the title compound, C_21_H_21_N_3_O_2_, (**I**), crystallizes in space group *Pna*2_1_, compared to *Pbca* for the first ortho­rhom­bic polymorph (**I^i^**) [Obasi *et al.* (2016 ▸). *J. Mol. Struct*. **1120**, 180–186]. The difference in the structure of the two polymorphs resides in the orientation of the 4-meth­oxy moiety of the (4-meth­oxy­phen­yl)allyl­idene unit with respect to the phenyl ring to which it is attached. Compound (**I**) also exhibits rotational disorder of the phenyl ring of the 4-amino­anti­pyrine moiety. In the crystal of (**I**), the mol­ecules are linked by C—H⋯O and C—H⋯N hydrogen bonds, forming a three-dimensional network. The conformations, Hirshfeld surfaces, and two-dimensional fingerprint plots of the two polymorphs and closely related structures are compared.

## Abstract 2

A second ortho­rhom­bic polymorph of the title Schiff base crystallizes in space group *Pna*2_1_, compared to *Pbca* for the first polymorph. The difference in the structure of the two polymorphs resides in the orientation of the 4-meth­oxy moiety of the (4-meth­oxy­phen­yl)allyl­idene unit.

## 1. Chemical context

4-Amino­anti­pyrine (C_11_H_13_N_3_O; 4-AAP) has been used to form a large number of Schiff base compounds by condensation with an aldehyde or a ketone. A search of the Cambridge Structural Database (CSD, Version 6.01, update November 2025; Groom *et al.*, 2016 ▸) for Schiff base organic compounds involving 4-AAP gave over 240 hits. The vast majority of such Schiff bases involve substituted benzaldehydes.

Aguilar-Llanos *et al.*, (2022 ▸; 2023 ▸) have described the synthesis of a number of Schiff base compounds involving cinnamaldehydes. As they explained, the use of such aldehydes results in the presence of an extensive double-bond conjugated system *via* the amino group of 4-AAP. These compounds were shown to have potential biological activity and could be useful in optical applications (Ani *et al.*, 2021 ▸; Arroudj *et al.*, 2016 ▸). The structure of the unsubstituted (phen­yl)prop-2-en-1-yl­idene derivative (**II**) (CSD refcode: FEVBUE) has been reported on by Li & Zhang (2005 ▸). More recently Aguilar-Llanos *et al.* (2023 ▸) have described the crystal structures, Hirshfeld surface analyses and biological activities of four Schiff base compounds involving various substituted cinnamaldehydes. They include the 4-AAP derivatives of [4-(di­methyl­amino)­phen­yl]prop-2-en-1-yl­idene (**III**: MODGUL) and (4-nitro­phen­yl)prop-2-en-1-yl­idene (**IV**: MODHEW).
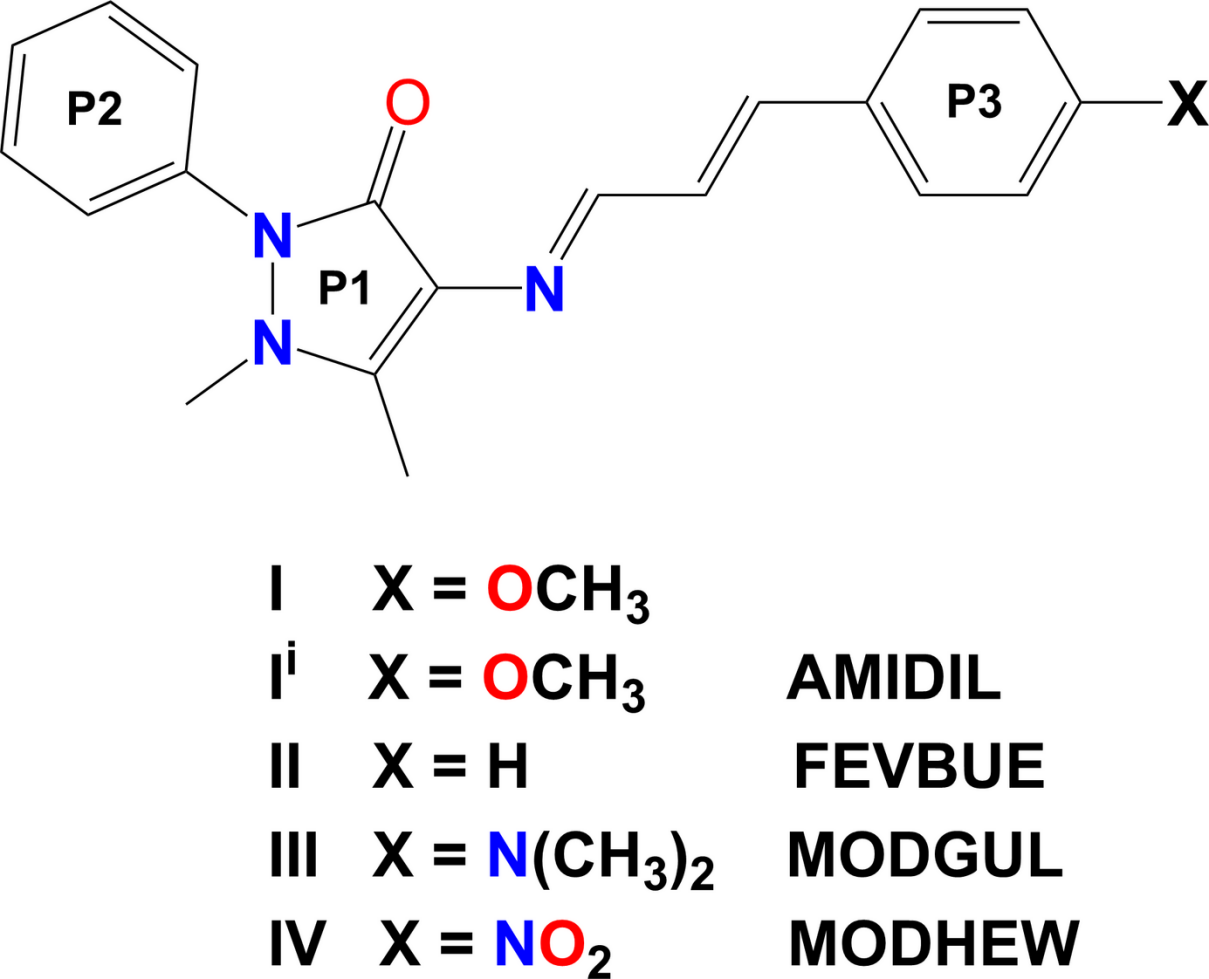

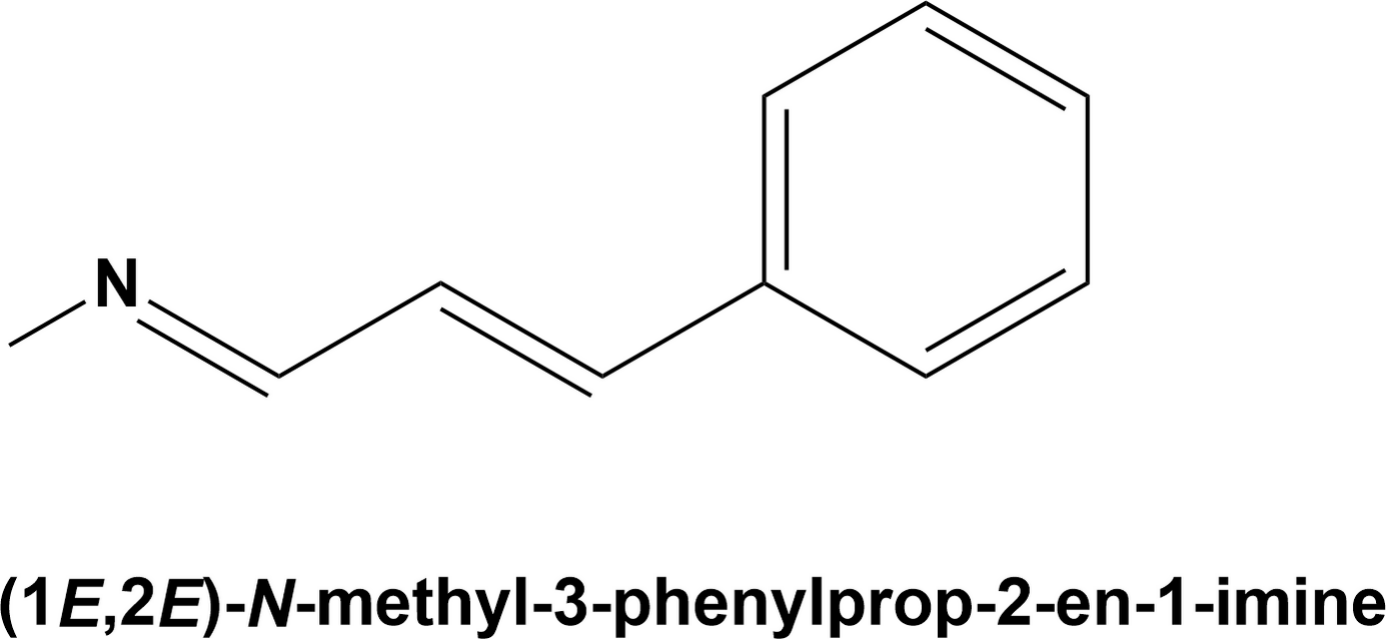


As part of our ongoing research into the chemistry of 4-AAP derivatives (Shankar *et al.*, 2023 ▸), we now describe the structure of a second ortho­rhom­bic polymorph of compound (**I**). The structure of this polymorph is compared to that of the first ortho­rhom­bic polymorph (**I^i^**), described by Obasi *et al.* (2016 ▸), and to that of compound (**II**), and in part to (**III**) and (**IV**).

## 2. Structural commentary

**Figure 1:**
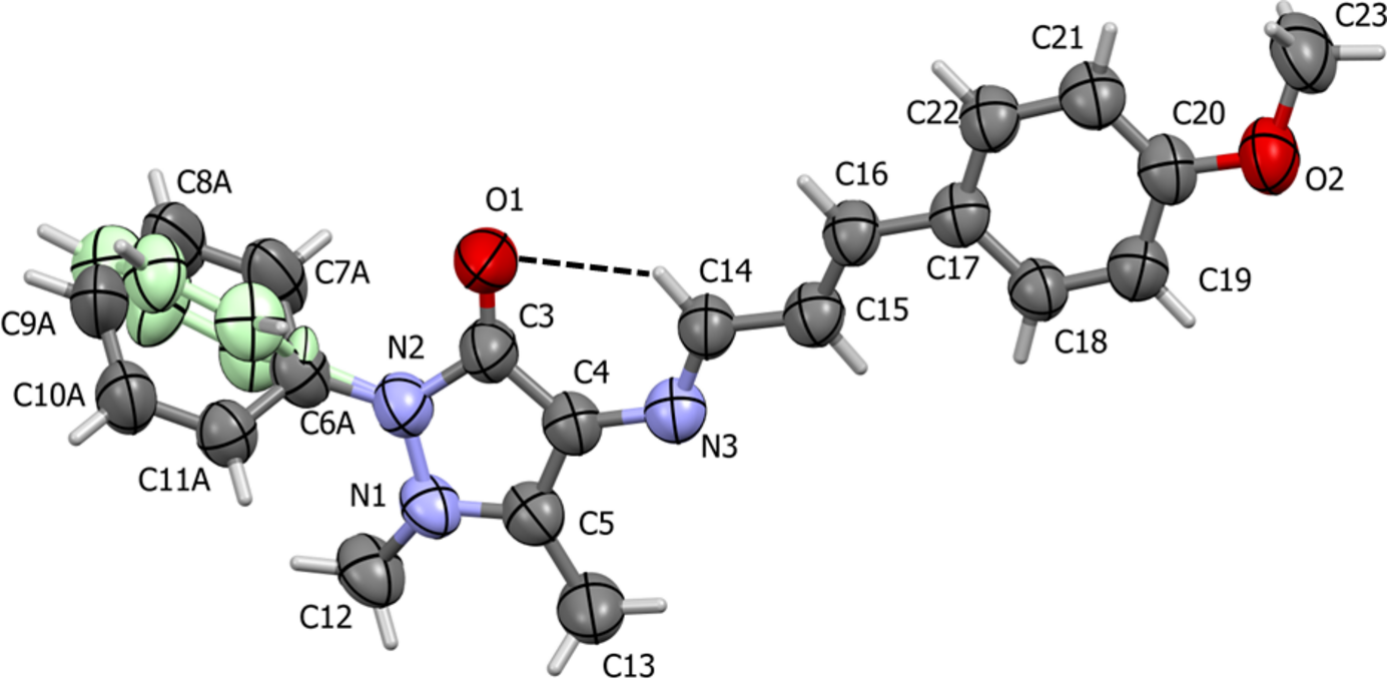
A view of the mol­ecular structure of the title compound, polymorph **I**, with displacement ellipsoids drawn at the 50% probability level. The minor component atoms of the positionally disordered phenyl ring (atoms C6*B*—C11*B*) are shown in pale green.

The title compound, C_21_H_21_N_3_O_2_ (**I**), was synthesized by the condensation of 4-amino­anti­pyrine with 3-(4-meth­oxy­phen­yl)acryl­aldehyde and single crystals were grown by recrystallization from a methanol solution. It crystallizes in the ortho­rhom­bic space group *Pna*2_1_. The mol­ecular structure of (**I**) is illustrated in Fig. 1 ▸. Selected geometrical parameters for (**I**) and related compounds are given in Table 1 ▸. An earlier report of the crystal structure of the title compound indicated that it had crystallized in the ortho­rhom­bic space group *Pbca* (**I^i^**) [CSD refcode AMIDa IL; Obasi *et al.*, 2016 ▸], which was also recrystallized from methanol solution. A comparison of calculated densities for (**I**) (1.241 g cm^−3^) and (**I^i^**) (1.257 g cm^−3^) suggests that the latter may be the more stable polymorph.

A significant feature of the crystal structure of (**I**) is the positional disorder of the 2-phenyl ring of 4-AAP, see Fig. 1 ▸. The occupancies of atoms C6–C11 and their attached H atoms disordered over two sets of sites were refined, giving an occupancy of 0.77 (1) for the major component (atoms C6*A*–C11*A*), and 0.23 (1) for the minor component (atoms C6*B*–C11*B*). The two rings were refined as regular hexa­gons and are inclined to each other by 68.5 (6)°. Such disorder in 4-AAP derivatives has been observed previously (Kant *et al.*, 2013 ▸; Tahir *et al.*, 2025 ▸).

**Figure 2:**
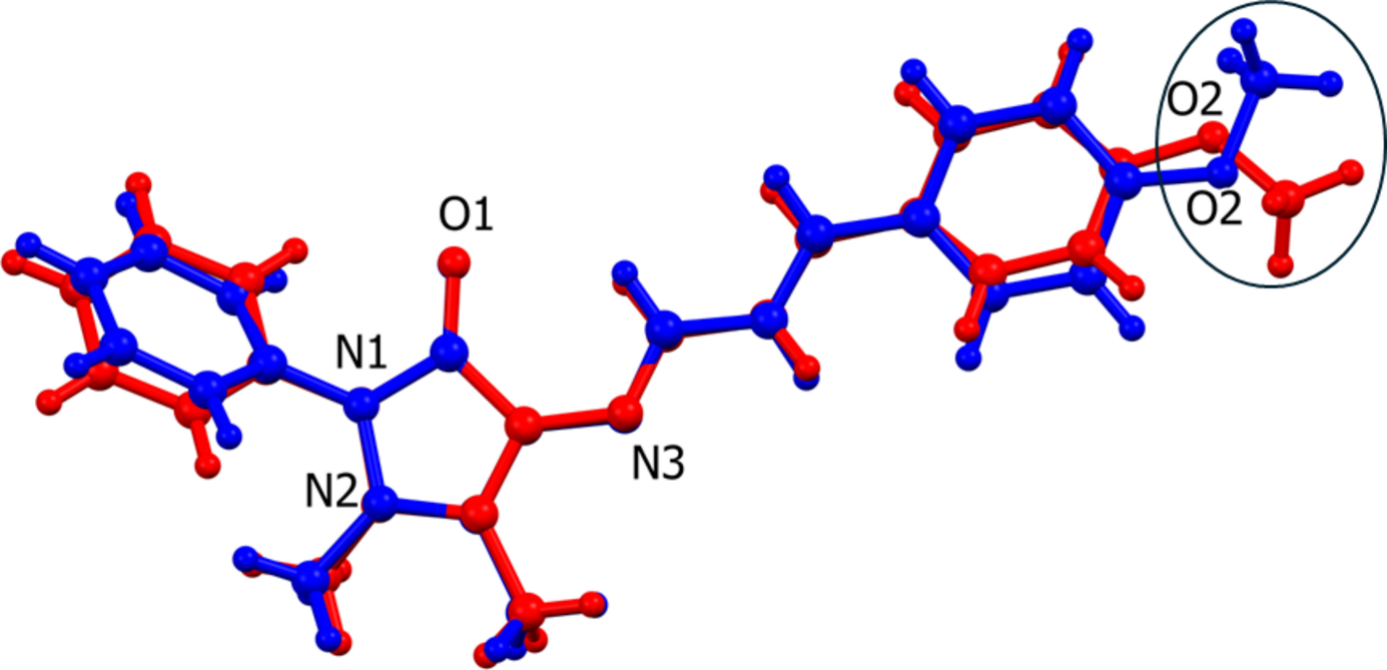
A view of the structural overlap of polymorph **I** (blue; major component, atoms C6*A–*C11*A*) and polymorph **I^i^** (red) [r.m.s. 0.137 Å; *Mercury* (Macrae *et al.*, 2020 ▸)].

The difference in the structure of the two polymorphs resides in the orientation of the 4-meth­oxy moiety (–O2—C23) of the (4-meth­oxy­phen­yl)allyl­idene unit with respect to ring P3 (C17–C22). This difference is illustrated in Fig. 2 ▸, a view of the structural overlap of polymorph (**I**) (major component of the 4-AAP phenyl ring, atoms C6*A*–C11*A*) and polymorph (**I^i^**). It can be seen that bond O2—C23 is *cis* with respect to the carbonyl bond C3=O1 in (**I**) but *trans* in (**I^i^**); see Fig. 2 ▸. The same group (atoms C20—O2—C23) is inclined to the mean plane of ring P3 (C17—C22) by 8.4 (5)° in (**I**) and 4.9 (2)° in (**I^i^**).

In the mol­ecule of (**I**), there is an intra­molecular C—H⋯O hydrogen bond forming an *S*(6) ring motif (C14—H14⋯O1; Table 2 ▸). The allyl­idine–amino chain (atoms N3 to C17) has an *E*,*E* configuration about bonds N3=C14 and C15=C16. This configuration is the same for all five structures. The N=C and C=C bond lengths are very similar in the two polymorphs, (**I**) and (**I^i^**). It can be seen that the N=C bond length varies from 1.282 (3) Å in (**IV**) to 1.294 (6) Å in (**I**). The C=C bond length [atoms C15=C16 in (**I**)] is 1.338 (6) Å, similar to the value in (**I^i^**) but slightly longer than the values for compounds (**II**), (**III**) and (**IV**).

An analysis using *Mercury* (Macrae *et al.*, 2020 ▸) of these two bond lengths was carried out for 87 compounds containing the (1*E*,2*E*)-*N*-methyl-3-phenyl­prop-2-en-1-imine moiety found in the CSD. It revealed that the N=C bond length varies from 1.256 to 1.316 Å, with an average value of 1.280 (15) Å (median value of 1.277 Å, skewness 0.877). The C=C bond length varies from 1.305 to 1.376 Å, with an average value of 1.334 (9) Å (median value of 1.333 Å, skewness 0.858). Hence, the values for all five compounds described here lie within these limits. The main difference is in the N=C bond length which is longer in (**I**) and (**I**^i^) compared to the values for (**II**), (**III**) and (**IV**) (Table 1 ▸).

**Figure 3:**
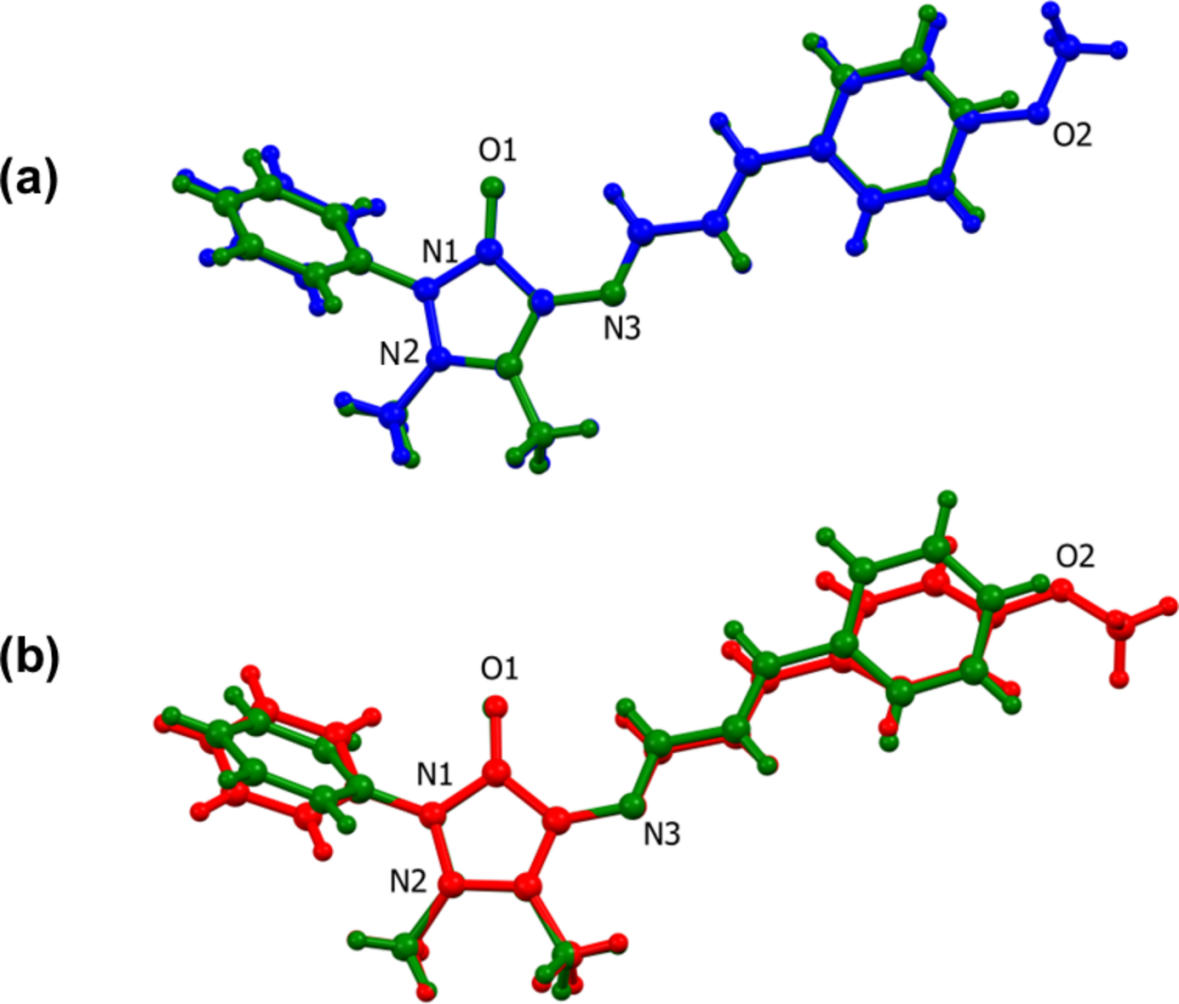
(*a*) A view of the structural overlap of **I** (blue; major component, atoms C6*A–*C11*A*) and **II** (green) [r.m.s. 0.0448 Å; *Mercury* (Macrae *et al.*, 2020 ▸)], (*b*) A view of the structural overlap of **I^i^** (red) and **II** (green) [r.m.s. 0.0637 Å; *Mercury* (Macrae *et al.*, 2020 ▸)].

Various dihedral angles in structures (**I**), (**I**^i^) (**II**), (**III**) and (**IV**) are compared in Table 1 ▸. The conformation of (**I**) is similar to that of compounds (**II**), (**III**) and (**IV**). Polymorph (**I^i^**) is the odd one out with for example, the P1 to P3 dihedral angle being the largest at 25.1 (1)°, compared to 2.1 (2)° in (**I**). A view of the structural overlap of (**I**) and (**II**) (Fig. 3 ▸*a*) and of (**I^i^**) and (**II**) (Fig. 3 ▸*b*) illustrates this situation.

## 3. Supra­molecular features

**Figure 4:**
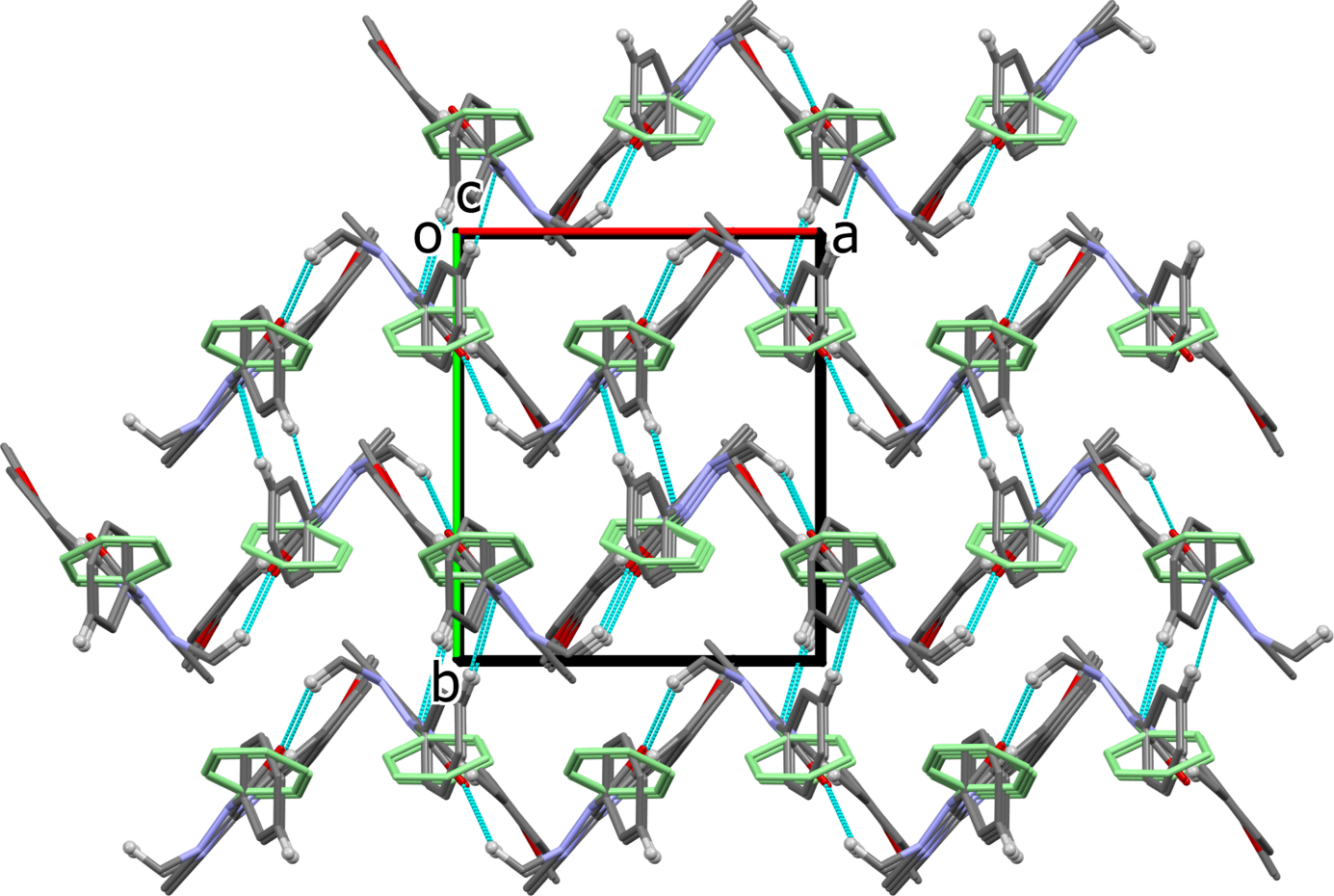
A view along the *c*-axis of the crystal packing of polymorph **I**, showing the hydrogen bonds as dashed lines (Table 2 ▸). The minor component atoms of the positionally disordered phenyl ring (atoms C6*B–*C11*B*) are shown in pale green. For clarity, only the H atoms (small white spheres) involved in hydrogen bonding have been included.

In the crystal of (**I**), the mol­ecules are linked by C—H⋯O hydrogen bonds (Table 2 ▸) forming zigzag chains propagating along the *a*-axis direction. The mol­ecules are also linked by C—H⋯N hydrogen bonds about the screw axis parallel to the *c*-axis direction. The combination of these non-classical hydrogen bonds results in the formation of a three-dimensional network, as shown in Fig. 4 ▸.

## 4. Hirshfeld surface analysis and two-dimensional fingerprint plots

**Figure 5:**
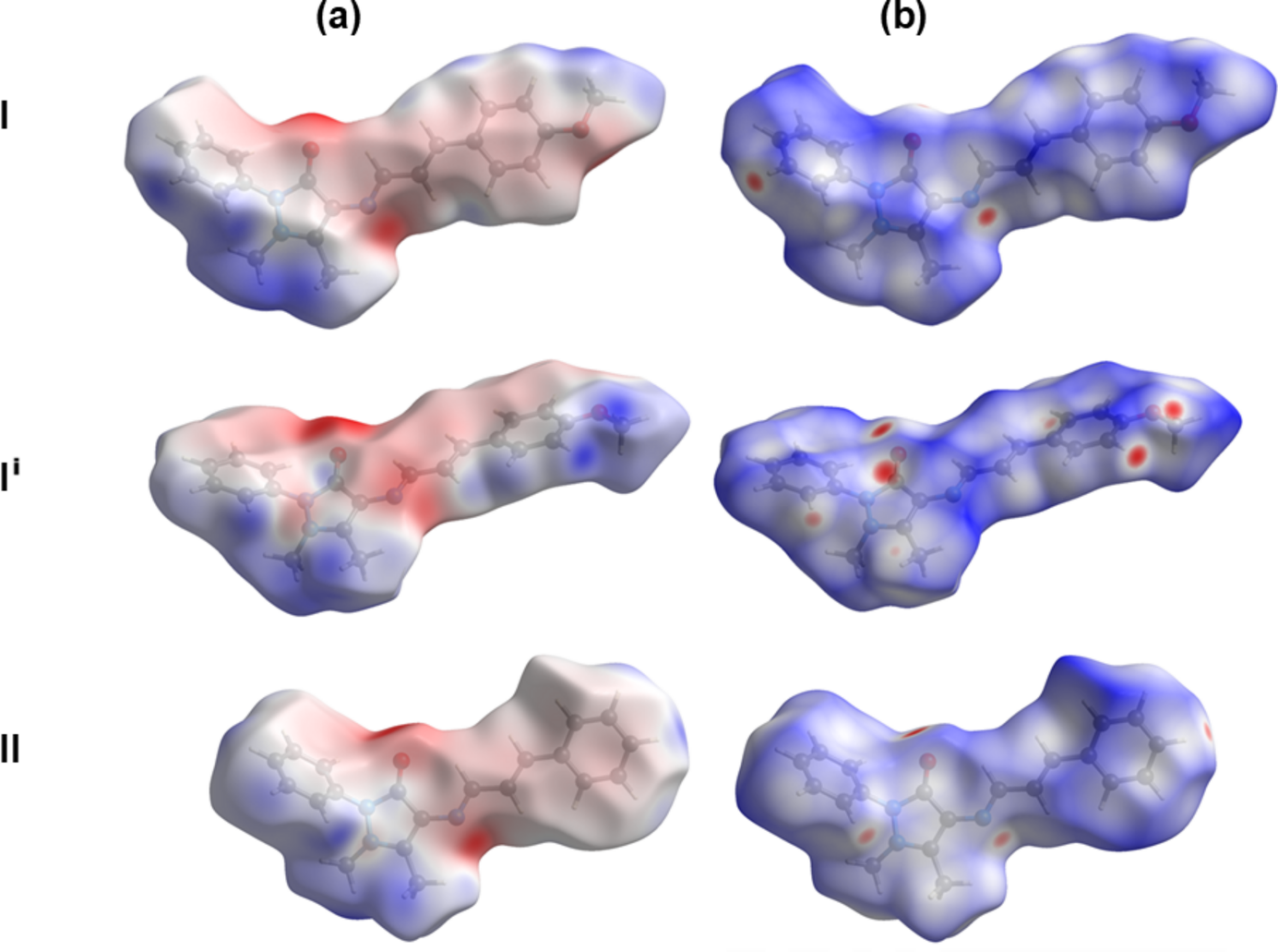
(*a*) The electrostatic potential surface of polymorphs **I** (major component, atoms C6*A*–C11*A*) and **I^i^** and compound **II**, mapped over colour ranges −0.09 to 0.05, −0.07 to 0.05, and −0.09 to 0.06 au., respectively; (*b*) the Hirshfeld surface of polymorphs **I** and **I^i^** and compound **II**, mapped over *d*_norm_ in the colour ranges −0.16 to 1.37, −0.21 to 1.28, and −0.30 to 1.91 au., respectively.

The Hirshfeld surface analyses and the associated two-dimensional fingerprint plots were performed with *CrystalExplorer17* (Spackman *et al.*, 2021 ▸), following the protocol of Tan *et al.* (2019 ▸). The electrostatic potential and Hirshfeld surfaces for polymorphs (**I**) and (**I^i^**) and compound (**II**) are illustrated in Fig. 5 ▸. For (**I**), ring P2 comprises the major component *viz.* atoms C6*A*–C11*A*. The presence of prominent red spots indicate that short contacts are particularly significant in the crystal packing of all three compounds. The various contributions to the HS of the short contacts in the crystals structures of all five compounds, (**I**), (**I**^i^) (**II**), (**III**) and (**IV**), are compared in Table S1 of the supporting information. As mentioned above, Aguilar-Llanos *et al.* (2023 ▸) have analysed the crystal structures and the Hirshfeld surfaces and two-dimensional fingerprint plots of compounds (**III**) and (**IV**).

**Figure 6:**
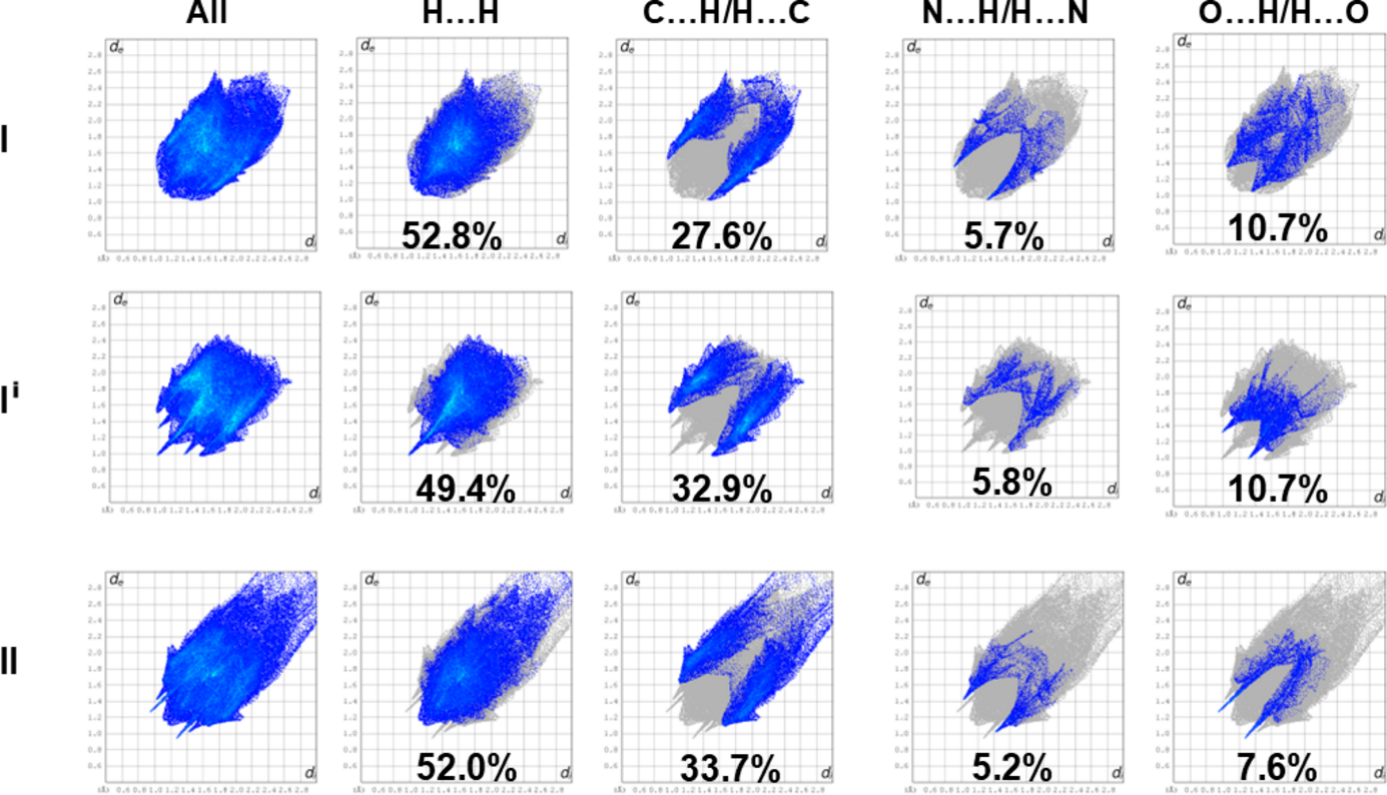
The full two-dimensional fingerprint plots for polymorphs **I** (major component, atoms C6*A*–C11*A*) and **I^i^** and compound **II**, and those delineated into H⋯H, C⋯H/H⋯C, N⋯H/H⋯N and O⋯H/H⋯O contacts.

The full two-dimensional fingerprint plots for (**I)**, **(I^i^**) and (**II**) are given in Fig. 6 ▸. In all three compounds the H⋯H contacts have a major contribution to the Hirshfeld surface, varying from 49.4% in (**I^i^**) to 52.8% in (**I**). The second most significant contribution is from the C⋯H/H⋯C contacts that vary from 27.6% in (**I**) to 33.7% in (**II**). The N⋯H/H⋯N contacts in (**I**) and (**I^i^**) are very similar 5.7 *cf*. 5.8%, while being 5.2% in (**II**). For (**I**) and (**II**), these contacts have sharp pincer-like peaks at *d_e_ + d_i_* ≃ 2.5 and 2.42 Å. respectively. The most significant difference concerns the contributions of the O⋯H/H⋯O contacts, which are 10.7% in (**I**) and (**I^i^**), while in the absence of the meth­oxy group in (**II**) it is only 7.6%. However, in (**II**) the O⋯H/H⋯O contacts have sharp pincer-like spikes at *d_e_ + d_i_* ≃ 2.2 Å. Similar sharp pincer-like spikes are also observed in (**I^i^**) but at a longer distance, *d_e_ + d_i_* ≃ 2.35Å. The C⋯C contacts contribute 1.4% in (**I**) but only 0.2% in (**I^i^**) and 0.7% in (**II**). Other contacts in general contribute less than 1%. The contributions of the various contacts can be correlated with the hydrogen bonds and other inter­atomic inter­actions in the crystal structures of the three compounds.

## 5. Synthesis

To a methano­lic solution of 4-amino­anti­pyrine (1 mmol), prepared in a round-bottom flask, 4-meth­oxy­cinnamaldehyde (1 mmol) dissolved in 20 ml of methanol was added dropwise under continuous stirring at room temperature. The reaction mixture was stirred for 15 min and subsequently heated under reflux for 8 h, to ensure completion of the condensation reaction. After reflux, the mixture was allowed to cool slowly to room temperature, leading to the formation of a brown precipitate. The resulting solid was collected by filtration and washed several times with cold methanol to remove unreacted starting materials and impurities, affording the desired product in pure form. Yellow prismatic crystals of (**I**) were obtained by dissolving the purified compound in methanol and allowing the solution to undergo slow evaporation at room temperature over a period of *ca.* 10 days. FTIR (KBr pellet, cm^−1^); 1602 C=N stretch, 1635 C=O stretch, 1570 C=C stretch, 1022 C—O stretch. UV/vis (ethanol solution, nm): 240, 359. For further spectroscopic details and TGA/DTA and second-harmonic generation studies, see the supporting information.

## 6. Refinement details

Crystal data, data collection and structure refinement details are summarized in Table 3 ▸. The C-bound H atoms were included in calculated positions and refined as riding atoms; C—H = 0.95–0.98 Å, *U*_iso_(H) = 1.5*U*_eq_(C-meth­yl) and 1.2*U*_eq_(C) for other H atoms. The occupancies of the positionally disordered 2-phenyl ring of 4-AAP were refined giving 0.77 (1) for the major component (atoms C6*A*–C11*A*), compared to 0.23 (1) for the minor component (atoms C6*B*–C11*B*). The two rings were refined as regular hexa­gons *i.e.* rigid groups with a C—C separation of 1.39 Å. The structure was refined as a two-component inversion twin [BASF = −0.26 (6); this value has no physical meaning].

## Supplementary Material

Crystal structure: contains datablock(s) I, Global. DOI: 10.1107/S2056989026001039/hb8194sup1.cif

Structure factors: contains datablock(s) I. DOI: 10.1107/S2056989026001039/hb8194Isup2.hkl

Table S1. Relative percentage contributions (%) of close contacts to the Hirshfeld surfaces of compounds I, Ii, II, III and IV. FTIR spectroscopic analysis of I. UV-Vis spectroscopic analysis of I. 1H NMR spectrum of I. Thermogravimetric analysis (TGA-black) and differential thermal analysis (DTA-blue) of I. Optical limiting behaviour of I. DOI: 10.1107/S2056989026001039/hb8194sup3.pdf

Supporting information file. DOI: 10.1107/S2056989026001039/hb8194Isup4.cml

CCDC reference: 2527948

Additional supporting information:  crystallographic information; 3D view; checkCIF report

## Figures and Tables

**Table 1:** Selected geometrical parameters (Å, °) for compounds (**I**), (**I^i^**), (**II**), (**III**) and (**IV**) For (**I**) (major component of ring P2, atoms C6*A*–C11*A*). Mean plane P1 (N1/N2/C3–C5), mean plane P2 (C6–C11) and mean plane P3 (C17–C22); see Scheme 1.

Bond	(**I**)	(**I^i*a*^**)	(**II^*b*^**)	(**III^*c*^**)	(**IV^*c*^**)
N=C	1.294 (6)	1.2908 (6)	1.2805 (3)	1.288 (3)	1.282 (3)
C=C	1.338 (6)	1.3388 (1)	1.3282 (3)	1.326 (3)	1.326 (4)
					
Dihedral angle	(**I**)	(**I^i*a*^**)	(**II^*b*^**)	(**III^*c*^**)	(**IV^*c*^**)
P1 to P2	49.8 (3)	33.0 (1)	54.7 (1)	48.4 (1)	54.1 (1)
P1 to P3	2.1 (2)	25.1 (1)	9.6 (1)	12.7 (1)	5.3 (1)
P2 to P3	48.7 (3)	21.3 (1)	61.9 (1)	56.7 (1)	59.3 (1)

References: (*a*) Obasi *et al.* (2016 ▸); (*b*) Li & Zhang (2005 ▸); (*c*) Aguilar-Llanos *et al.* (2023 ▸).

**Table 2:** Hydrogen-bond geometry (Å, °)

*D*—H⋯*A*	*D*—H	H⋯*A*	*D*⋯*A*	*D*—H⋯*A*
C14—H14⋯O1	0.94	2.35	3.020 (6)	128
C12—H12*C*⋯O1^i^	0.97	2.50	3.232 (8)	133
C10*A*—H10*A*⋯N3^ii^	0.94	2.58	3.511 (6)	173

Symmetry codes: (i) 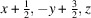; (ii) 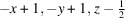.

**Table 3:** Experimental details

Crystal data	
Chemical formula	C_21_H_21_N_3_O_2_
*M*_r_	347.41
Crystal system, space group	Orthorhombic, *Pna*2_1_
Temperature (K)	250
*a*, *b*, *c* (Å)	9.4079 (4), 11.0918 (5), 17.8194 (10)
*V* (Å^3^)	1859.46 (16)
*Z*	4
Radiation type	Cu *K*α
μ (mm^−1^)	0.65
Crystal size (mm)	0.47 × 0.38 × 0.24

Data collection	
Diffractometer	Stoe Stadivari
Absorption correction	Analytical (*X-RED32* and *LANA*; Stoe, 2024 ▸)
*T*_min_, *T*_max_	0.713, 0.841
No. of measured, independent and observed [*I* > 2σ(*I*)] reflections	14750, 3050, 2818
*R*_int_	0.049
(sin θ/λ)_max_ (Å^−1^)	0.596

Refinement	
*R*[*F*^2^ > 2σ(*F*^2^)], *wR*(*F*^2^), *S*	0.067, 0.190, 1.10
No. of reflections	3050
No. of parameters	270
No. of restraints	1
H-atom treatment	H-atom parameters constrained
Δρ_max_, Δρ_min_ (e Å^−3^)	0.25, −0.22
Absolute structure	Refined as an inversion twin
Absolute structure parameter	−0.2 (6)

Computer programs: *X-AREAPilatus3_SV*, *Recipe*, *Integrate* and *LANA* (Stoe, 2024 ▸), *SHELXT2019/3* (Sheldrick, 2015*a* ▸), *SHELXL2019/3* (Sheldrick, 2015*b* ▸), *PLATON* (Spek, 2020 ▸), *Mercury* (Macrae *et al.*, 2020 ▸) and *publCIF* (Westrip, 2010 ▸).

## supplementary crystallographic information

### 4-{[(1E,2E)-3-(4-Methoxyphenyl)prop-2-en-1-ylidene]amino}-1,5-dimethyl-2-phenyl-1H-pyrazol-3(2H)-one Crystal data

**Table:**

C_21_H_21_N_3_O_2_	*D*_x_ = 1.241 Mg m^−^^3^
*M**_r_* = 347.41	Cu *K*α radiation, λ = 1.54186 Å
Orthorhombic, *P**n**a*2_1_	Cell parameters from 24601 reflections
*a* = 9.4079 (4) Å	θ = 4.0–66.6°
*b* = 11.0918 (5) Å	µ = 0.65 mm^−^^1^
*c* = 17.8194 (10) Å	*T* = 250 K
*V* = 1859.46 (16) Å^3^	Prism, yellow
*Z* = 4	0.47 × 0.38 × 0.24 mm
*F*(000) = 736	

### 4-{[(1E,2E)-3-(4-Methoxyphenyl)prop-2-en-1-ylidene]amino}-1,5-dimethyl-2-phenyl-1H-pyrazol-3(2H)-one Data collection

**Table:**

Stoe Stadivari diffractometer	3050 independent reflections
Radiation source: Primux 100 micro	2818 reflections with *I* > 2σ(*I*)
Graded multilayer mirror monochromator	*R*_int_ = 0.049
Detector resolution: 5.81 pixels mm^-1^	θ_max_ = 66.8°, θ_min_ = 4.7°
rotation method, ω scans	*h* = −7→11
Absorption correction: analytical (X-Red32 and *LANA*; Stoe, 2024)	*k* = −13→12
*T*_min_ = 0.713, *T*_max_ = 0.841	*l* = −21→18
14750 measured reflections	

### 4-{[(1E,2E)-3-(4-Methoxyphenyl)prop-2-en-1-ylidene]amino}-1,5-dimethyl-2-phenyl-1H-pyrazol-3(2H)-one Refinement

**Table:**

Refinement on *F*^2^	Secondary atom site location: difference Fourier map
Least-squares matrix: full	Hydrogen site location: inferred from neighbouring sites
*R*[*F*^2^ > 2σ(*F*^2^)] = 0.067	H-atom parameters constrained
*wR*(*F*^2^) = 0.190	*w* = 1/[σ^2^(*F*_o_^2^) + (0.1392*P*)^2^ + 0.1581*P*] where *P* = (*F*_o_^2^ + 2*F*_c_^2^)/3
*S* = 1.10	(Δ/σ)_max_ < 0.001
3050 reflections	Δρ_max_ = 0.25 e Å^−^^3^
270 parameters	Δρ_min_ = −0.22 e Å^−^^3^
1 restraint	Absolute structure: Refined as an inversion twin
Primary atom site location: dual	Absolute structure parameter: −0.2 (6)

### 4-{[(1E,2E)-3-(4-Methoxyphenyl)prop-2-en-1-ylidene]amino}-1,5-dimethyl-2-phenyl-1H-pyrazol-3(2H)-one Special details

**Table:**

Geometry. All esds (except the esd in the dihedral angle between two l.s. planes) are estimated using the full covariance matrix. The cell esds are taken into account individually in the estimation of esds in distances, angles and torsion angles; correlations between esds in cell parameters are only used when they are defined by crystal symmetry. An approximate (isotropic) treatment of cell esds is used for estimating esds involving l.s. planes.
Refinement. Refined as a 2-component inversion twin.

### 4-{[(1E,2E)-3-(4-Methoxyphenyl)prop-2-en-1-ylidene]amino}-1,5-dimethyl-2-phenyl-1H-pyrazol-3(2H)-one Fractional atomic coordinates and isotropic or equivalent isotropic displacement parameters (Å^2^)

**Table:**

	*x*	*y*	*z*	*U*_iso_*/*U*_eq_	Occ. (<1)
O1	0.4835 (6)	0.7889 (4)	0.3359 (2)	0.1123 (15)	
O2	0.3128 (4)	0.8986 (4)	0.9176 (2)	0.0900 (10)	
N1	0.7078 (4)	0.5527 (3)	0.2924 (2)	0.0703 (10)	
N2	0.6255 (4)	0.6510 (4)	0.2727 (2)	0.0762 (10)	
N3	0.6049 (4)	0.6491 (3)	0.4744 (2)	0.0685 (9)	
C3	0.5660 (5)	0.7032 (4)	0.3371 (3)	0.0756 (11)	
C4	0.6249 (4)	0.6350 (4)	0.3980 (2)	0.0639 (9)	
C5	0.7130 (4)	0.5493 (4)	0.3678 (3)	0.0698 (11)	
C6A	0.5847 (6)	0.6725 (5)	0.1993 (2)	0.071 (4)	0.766 (9)
C7A	0.5829 (8)	0.7907 (4)	0.1734 (3)	0.102 (3)	0.766 (9)
H7A	0.614095	0.853680	0.204576	0.122*	0.766 (9)
C8A	0.5348 (9)	0.8157 (3)	0.1013 (3)	0.130 (5)	0.766 (9)
H8A	0.533573	0.895605	0.083709	0.156*	0.766 (9)
C9A	0.4887 (9)	0.7226 (4)	0.0551 (3)	0.120 (4)	0.766 (9)
H9A	0.456169	0.739466	0.006371	0.144*	0.766 (9)
C10A	0.4905 (6)	0.6044 (4)	0.0811 (3)	0.090 (2)	0.766 (9)
H10A	0.459288	0.541400	0.049899	0.108*	0.766 (9)
C11A	0.5386 (6)	0.5794 (3)	0.1532 (3)	0.0718 (15)	0.766 (9)
H11A	0.539810	0.499473	0.170765	0.086*	0.766 (9)
C6B	0.5808 (14)	0.6869 (16)	0.1980 (6)	0.056 (10)	0.234 (9)
C7B	0.4422 (12)	0.6887 (16)	0.1711 (7)	0.079 (6)	0.234 (9)
H7B	0.367754	0.658140	0.200724	0.095*	0.234 (9)
C8B	0.4136 (11)	0.7357 (16)	0.1004 (8)	0.086 (6)	0.234 (9)
H8B	0.319782	0.736937	0.082192	0.103*	0.234 (9)
C9B	0.5236 (15)	0.7809 (18)	0.0566 (8)	0.091 (9)	0.234 (9)
H9B	0.504239	0.812671	0.008769	0.109*	0.234 (9)
C10B	0.6623 (13)	0.7791 (17)	0.0835 (8)	0.097 (8)	0.234 (9)
H10B	0.736669	0.809607	0.053878	0.116*	0.234 (9)
C11B	0.6909 (11)	0.7320 (16)	0.1542 (8)	0.080 (6)	0.234 (9)
H11B	0.784645	0.730810	0.172410	0.096*	0.234 (9)
C12	0.8165 (5)	0.5041 (6)	0.2436 (4)	0.0872 (14)	
H12A	0.779970	0.498881	0.192791	0.131*	
H12B	0.843321	0.424322	0.260830	0.131*	
H12C	0.899041	0.556477	0.244410	0.131*	
C13	0.8049 (6)	0.4635 (6)	0.4089 (4)	0.0947 (16)	
H13C	0.776602	0.461012	0.461201	0.142*	
H13B	0.903133	0.489498	0.405302	0.142*	
H13A	0.795296	0.383807	0.387074	0.142*	
C14	0.5131 (5)	0.7256 (4)	0.5000 (3)	0.0684 (10)	
H14	0.459088	0.772354	0.466572	0.082*	
C15	0.4937 (5)	0.7391 (4)	0.5792 (3)	0.0706 (11)	
H15	0.553421	0.694939	0.611326	0.085*	
C16	0.3955 (5)	0.8107 (4)	0.6102 (3)	0.0680 (10)	
H16	0.336416	0.853271	0.576916	0.082*	
C17	0.3704 (4)	0.8300 (4)	0.6898 (3)	0.0643 (10)	
C18	0.4329 (5)	0.7606 (4)	0.7469 (3)	0.0679 (10)	
H18	0.492218	0.695758	0.733660	0.081*	
C19	0.4103 (5)	0.7843 (4)	0.8206 (3)	0.0726 (11)	
H19	0.453081	0.735269	0.857201	0.087*	
C20	0.3238 (4)	0.8809 (4)	0.8430 (3)	0.0694 (10)	
C21	0.2578 (5)	0.9483 (5)	0.7886 (3)	0.0756 (12)	
H21	0.196974	1.011891	0.802261	0.091*	
C22	0.2811 (5)	0.9224 (4)	0.7131 (3)	0.0722 (11)	
H22	0.234755	0.969173	0.676519	0.087*	
C23	0.2406 (8)	1.0053 (7)	0.9419 (4)	0.108 (2)	
H23A	0.286399	1.075518	0.920300	0.162*	
H23B	0.244078	1.010390	0.996189	0.162*	
H23C	0.142273	1.002178	0.925572	0.162*	

### 4-{[(1E,2E)-3-(4-Methoxyphenyl)prop-2-en-1-ylidene]amino}-1,5-dimethyl-2-phenyl-1H-pyrazol-3(2H)-one Atomic displacement parameters (Å^2^)

**Table:**

	*U*^11^	*U*^22^	*U*^33^	*U*^12^	*U*^13^	*U*^23^
O1	0.149 (4)	0.119 (2)	0.069 (2)	0.066 (3)	0.005 (2)	−0.003 (2)
O2	0.087 (2)	0.120 (3)	0.063 (2)	0.0125 (19)	0.0072 (15)	−0.0088 (18)
N1	0.0553 (18)	0.086 (2)	0.070 (2)	0.0112 (15)	0.0025 (15)	−0.0163 (17)
N2	0.078 (2)	0.087 (2)	0.063 (3)	0.0124 (18)	0.0048 (18)	−0.0074 (17)
N3	0.0570 (19)	0.082 (2)	0.067 (2)	0.0018 (15)	−0.0002 (15)	−0.0026 (16)
C3	0.079 (3)	0.088 (2)	0.059 (3)	0.016 (2)	0.004 (2)	−0.003 (2)
C4	0.055 (2)	0.075 (2)	0.062 (2)	−0.0016 (16)	0.0017 (16)	−0.0056 (17)
C5	0.055 (2)	0.080 (2)	0.074 (3)	0.0029 (17)	−0.0045 (18)	−0.012 (2)
C6A	0.072 (8)	0.074 (5)	0.067 (9)	−0.011 (4)	0.017 (6)	−0.006 (4)
C7A	0.157 (9)	0.076 (4)	0.073 (4)	−0.032 (4)	0.025 (4)	−0.010 (3)
C8A	0.240 (15)	0.083 (5)	0.067 (5)	−0.010 (6)	0.024 (6)	0.007 (4)
C9A	0.204 (13)	0.098 (6)	0.058 (5)	0.020 (7)	−0.009 (6)	−0.004 (4)
C10A	0.110 (5)	0.092 (4)	0.067 (4)	−0.005 (4)	−0.007 (3)	−0.010 (3)
C11A	0.073 (3)	0.074 (3)	0.068 (4)	−0.007 (2)	0.004 (3)	−0.003 (2)
C6B	0.06 (2)	0.082 (18)	0.027 (17)	−0.008 (13)	−0.012 (14)	0.003 (11)
C7B	0.045 (9)	0.107 (14)	0.085 (14)	−0.015 (8)	0.013 (8)	0.001 (11)
C8B	0.068 (12)	0.114 (16)	0.076 (15)	−0.004 (11)	−0.017 (10)	−0.009 (12)
C9B	0.064 (13)	0.102 (19)	0.11 (2)	−0.011 (12)	−0.027 (13)	−0.004 (17)
C10B	0.082 (14)	0.14 (2)	0.072 (15)	−0.008 (14)	−0.001 (11)	0.027 (13)
C11B	0.053 (10)	0.104 (14)	0.084 (14)	−0.023 (9)	0.006 (8)	0.004 (11)
C12	0.066 (3)	0.110 (3)	0.085 (3)	0.012 (2)	0.012 (2)	−0.020 (3)
C13	0.083 (3)	0.118 (4)	0.084 (4)	0.032 (3)	−0.008 (2)	−0.011 (3)
C14	0.064 (2)	0.083 (2)	0.058 (2)	0.0054 (19)	−0.0010 (17)	−0.0024 (19)
C15	0.060 (2)	0.084 (2)	0.068 (3)	0.0046 (18)	−0.0050 (17)	−0.0026 (19)
C16	0.058 (2)	0.085 (2)	0.061 (2)	0.0032 (17)	−0.0047 (17)	−0.0020 (19)
C17	0.0499 (18)	0.077 (2)	0.066 (3)	−0.0021 (15)	−0.0020 (17)	−0.0028 (18)
C18	0.066 (2)	0.074 (2)	0.064 (2)	0.0031 (18)	−0.0038 (18)	−0.0079 (19)
C19	0.071 (2)	0.081 (2)	0.066 (3)	0.0005 (19)	−0.002 (2)	0.0011 (19)
C20	0.056 (2)	0.089 (2)	0.063 (3)	−0.0050 (18)	0.0051 (16)	−0.006 (2)
C21	0.055 (2)	0.095 (3)	0.077 (3)	0.0094 (19)	0.0006 (19)	−0.008 (2)
C22	0.057 (2)	0.091 (3)	0.069 (3)	0.0066 (18)	−0.0058 (18)	−0.003 (2)
C23	0.108 (5)	0.137 (5)	0.079 (4)	0.024 (4)	0.013 (3)	−0.027 (3)

### 4-{[(1E,2E)-3-(4-Methoxyphenyl)prop-2-en-1-ylidene]amino}-1,5-dimethyl-2-phenyl-1H-pyrazol-3(2H)-one Geometric parameters (Å, º)

**Table:**

O1—C3	1.227 (6)	C9B—C10B	1.3900
O2—C20	1.349 (6)	C9B—H9B	0.9400
O2—C23	1.431 (7)	C10B—C11B	1.3900
N1—C5	1.344 (6)	C10B—H10B	0.9400
N1—N2	1.382 (6)	C11B—H11B	0.9400
N1—C12	1.447 (6)	C12—H12A	0.9700
N2—C6A	1.383 (5)	C12—H12B	0.9700
N2—C3	1.402 (6)	C12—H12C	0.9700
N2—C6B	1.451 (10)	C13—H13C	0.9700
N3—C14	1.294 (6)	C13—H13B	0.9700
N3—C4	1.382 (6)	C13—H13A	0.9700
C3—C4	1.435 (7)	C14—C15	1.430 (6)
C4—C5	1.372 (6)	C14—H14	0.9400
C5—C13	1.479 (7)	C15—C16	1.338 (6)
C6A—C7A	1.3900	C15—H15	0.9400
C6A—C11A	1.3900	C16—C17	1.454 (6)
C7A—C8A	1.3900	C16—H16	0.9400
C7A—H7A	0.9400	C17—C22	1.388 (6)
C8A—C9A	1.3900	C17—C18	1.405 (6)
C8A—H8A	0.9400	C18—C19	1.355 (7)
C9A—C10A	1.3900	C18—H18	0.9400
C9A—H9A	0.9400	C19—C20	1.403 (6)
C10A—C11A	1.3900	C19—H19	0.9400
C10A—H10A	0.9400	C20—C21	1.373 (7)
C11A—H11A	0.9400	C21—C22	1.394 (7)
C6B—C7B	1.3900	C21—H21	0.9400
C6B—C11B	1.3900	C22—H22	0.9400
C7B—C8B	1.3900	C23—H23A	0.9700
C7B—H7B	0.9400	C23—H23B	0.9700
C8B—C9B	1.3900	C23—H23C	0.9700
C8B—H8B	0.9400		
			
C20—O2—C23	117.0 (4)	C9B—C10B—H10B	120.0
C5—N1—N2	107.3 (3)	C11B—C10B—H10B	120.0
C5—N1—C12	124.4 (4)	C10B—C11B—C6B	120.0
N2—N1—C12	122.4 (4)	C10B—C11B—H11B	120.0
N1—N2—C6A	122.2 (4)	C6B—C11B—H11B	120.0
N1—N2—C3	109.9 (4)	N1—C12—H12A	109.5
C6A—N2—C3	126.2 (4)	N1—C12—H12B	109.5
N1—N2—C6B	127.8 (8)	H12A—C12—H12B	109.5
C3—N2—C6B	121.4 (8)	N1—C12—H12C	109.5
C14—N3—C4	120.8 (4)	H12A—C12—H12C	109.5
O1—C3—N2	123.9 (5)	H12B—C12—H12C	109.5
O1—C3—C4	131.7 (4)	C5—C13—H13C	109.5
N2—C3—C4	104.4 (4)	C5—C13—H13B	109.5
C5—C4—N3	123.1 (4)	H13C—C13—H13B	109.5
C5—C4—C3	107.5 (4)	C5—C13—H13A	109.5
N3—C4—C3	129.3 (4)	H13C—C13—H13A	109.5
N1—C5—C4	110.5 (4)	H13B—C13—H13A	109.5
N1—C5—C13	122.2 (4)	N3—C14—C15	120.2 (4)
C4—C5—C13	127.2 (5)	N3—C14—H14	119.9
N2—C6A—C7A	118.8 (4)	C15—C14—H14	119.9
N2—C6A—C11A	121.1 (4)	C16—C15—C14	123.9 (4)
C7A—C6A—C11A	120.0	C16—C15—H15	118.0
C6A—C7A—C8A	120.0	C14—C15—H15	118.0
C6A—C7A—H7A	120.0	C15—C16—C17	127.1 (4)
C8A—C7A—H7A	120.0	C15—C16—H16	116.5
C9A—C8A—C7A	120.0	C17—C16—H16	116.5
C9A—C8A—H8A	120.0	C22—C17—C18	116.2 (4)
C7A—C8A—H8A	120.0	C22—C17—C16	119.8 (4)
C8A—C9A—C10A	120.0	C18—C17—C16	123.9 (4)
C8A—C9A—H9A	120.0	C19—C18—C17	121.9 (4)
C10A—C9A—H9A	120.0	C19—C18—H18	119.0
C11A—C10A—C9A	120.0	C17—C18—H18	119.0
C11A—C10A—H10A	120.0	C18—C19—C20	121.0 (5)
C9A—C10A—H10A	120.0	C18—C19—H19	119.5
C10A—C11A—C6A	120.0	C20—C19—H19	119.5
C10A—C11A—H11A	120.0	O2—C20—C21	125.6 (4)
C6A—C11A—H11A	120.0	O2—C20—C19	115.9 (4)
C7B—C6B—C11B	120.0	C21—C20—C19	118.5 (5)
C7B—C6B—N2	126.3 (9)	C20—C21—C22	119.8 (4)
C11B—C6B—N2	113.5 (8)	C20—C21—H21	120.1
C8B—C7B—C6B	120.0	C22—C21—H21	120.1
C8B—C7B—H7B	120.0	C17—C22—C21	122.4 (4)
C6B—C7B—H7B	120.0	C17—C22—H22	118.8
C7B—C8B—C9B	120.0	C21—C22—H22	118.8
C7B—C8B—H8B	120.0	O2—C23—H23A	109.5
C9B—C8B—H8B	120.0	O2—C23—H23B	109.5
C10B—C9B—C8B	120.0	H23A—C23—H23B	109.5
C10B—C9B—H9B	120.0	O2—C23—H23C	109.5
C8B—C9B—H9B	120.0	H23A—C23—H23C	109.5
C9B—C10B—C11B	120.0	H23B—C23—H23C	109.5
			
C5—N1—N2—C6A	−172.2 (4)	C8A—C9A—C10A—C11A	0.0
C12—N1—N2—C6A	33.8 (7)	C9A—C10A—C11A—C6A	0.0
C5—N1—N2—C3	−6.0 (5)	N2—C6A—C11A—C10A	176.3 (6)
C12—N1—N2—C3	−160.0 (5)	C7A—C6A—C11A—C10A	0.0
C5—N1—N2—C6B	−175.2 (8)	N1—N2—C6B—C7B	114.3 (11)
C12—N1—N2—C6B	30.8 (9)	C3—N2—C6B—C7B	−53.7 (14)
N1—N2—C3—O1	−176.9 (5)	N1—N2—C6B—C11B	−71.2 (11)
C6A—N2—C3—O1	−11.4 (8)	C3—N2—C6B—C11B	120.8 (9)
C6B—N2—C3—O1	−7.0 (10)	C11B—C6B—C7B—C8B	0.0
N1—N2—C3—C4	3.6 (5)	N2—C6B—C7B—C8B	174.1 (17)
C6A—N2—C3—C4	169.1 (5)	C6B—C7B—C8B—C9B	0.0
C6B—N2—C3—C4	173.6 (7)	C7B—C8B—C9B—C10B	0.0
C14—N3—C4—C5	−175.1 (4)	C8B—C9B—C10B—C11B	0.0
C14—N3—C4—C3	6.7 (7)	C9B—C10B—C11B—C6B	0.0
O1—C3—C4—C5	−179.3 (6)	C7B—C6B—C11B—C10B	0.0
N2—C3—C4—C5	0.1 (5)	N2—C6B—C11B—C10B	−174.8 (15)
O1—C3—C4—N3	−0.8 (9)	C4—N3—C14—C15	180.0 (4)
N2—C3—C4—N3	178.6 (4)	N3—C14—C15—C16	−176.0 (4)
N2—N1—C5—C4	6.1 (5)	C14—C15—C16—C17	−179.3 (4)
C12—N1—C5—C4	159.4 (4)	C15—C16—C17—C22	169.0 (4)
N2—N1—C5—C13	−173.2 (5)	C15—C16—C17—C18	−10.3 (7)
C12—N1—C5—C13	−19.8 (8)	C22—C17—C18—C19	−1.6 (6)
N3—C4—C5—N1	177.6 (4)	C16—C17—C18—C19	177.7 (4)
C3—C4—C5—N1	−3.8 (5)	C17—C18—C19—C20	−0.7 (7)
N3—C4—C5—C13	−3.2 (7)	C23—O2—C20—C21	−8.1 (7)
C3—C4—C5—C13	175.4 (5)	C23—O2—C20—C19	172.2 (5)
N1—N2—C6A—C7A	−141.3 (4)	C18—C19—C20—O2	−177.7 (4)
C3—N2—C6A—C7A	54.9 (6)	C18—C19—C20—C21	2.6 (6)
N1—N2—C6A—C11A	42.3 (6)	O2—C20—C21—C22	178.3 (4)
C3—N2—C6A—C11A	−121.5 (5)	C19—C20—C21—C22	−2.0 (6)
N2—C6A—C7A—C8A	−176.4 (5)	C18—C17—C22—C21	2.1 (6)
C11A—C6A—C7A—C8A	0.0	C16—C17—C22—C21	−177.2 (4)
C6A—C7A—C8A—C9A	0.0	C20—C21—C22—C17	−0.3 (7)
C7A—C8A—C9A—C10A	0.0		

### 4-{[(1E,2E)-3-(4-Methoxyphenyl)prop-2-en-1-ylidene]amino}-1,5-dimethyl-2-phenyl-1H-pyrazol-3(2H)-one Hydrogen-bond geometry (Å, º)

**Table:**

*D*—H···*A*	*D*—H	H···*A*	*D*···*A*	*D*—H···*A*
C14—H14···O1	0.94	2.35	3.020 (6)	128
C12—H12*C*···O1^i^	0.97	2.50	3.232 (8)	133
C10*A*—H10*A*···N3^ii^	0.94	2.58	3.511 (6)	173

Symmetry codes: (i) *x*+1/2, −*y*+3/2, *z*; (ii) −*x*+1, −*y*+1, *z*−1/2.
