# Supplementary material for: A second ortho­rhom­bic polymorph of 4-{[(1E,2E)-3-(4-meth­oxy­phen­yl)prop-2-en-1-yl­idene]amino}-1,5-dimethyl-2-phenyl-1H-pyrazol-3(2H)-one
Source: Acta Crystallogr E Crystallogr Commun. 2026 Feb 5;82(Pt 3):254–8. doi: 10.1107/S2056989026001039 (PMC12961671; doi:10.1107/S2056989026001039)
Supplement: Supplementary file 3 [file e-82-00254-sup3.pdf]

**Table S1. Relative percentage contributions (%) of close contacts to the Hirshfeld surfaces of compounds I, I$^{i}$, II, III and IV.**

**I (major component of ring B, atoms C6A-C11A)**

| | I | I$^{i}$ a) | II b) | III c) | IV c) |
|---|---|---|---|---|---|
| X | $OCH_3$ | $OCH_3$ | H | $N(CH_3)_2$ | $NO_2$ |
| Contact | | | Percentage contributions | | |
| H···H | 52.8 | 49.4 | 52.0 | 59.5 | 43.1 |
| C···H/H···C | 27.6 | 32.9 | 33.7 | 26.3 | 20.6 |
| N···H/H···N | 5.7 | 5.8 | 5.2 | 5.6 | 3.6 |
| O···H/H···O | 10.7 | 10.7 | 7.6 | 4.8 | 22.0 |
| C···C | 1.4 | 0.2 | 0.7 | 1.8 | 4.6 |
| N···C/C···N | 0 | 0 | 0.3 | 0.3 | 2.2 |
| O···C/C···O | 1.1 | 0.5 | 0.7 | 0.7 | 2.3 |

a) AMIDIL: Obasi *et al.*, (2016). J. Mol. Struc. 1120, 180-186, b) FEVBUE: Li & Zhang, (2005). Acta Cryst. E61, o375-0377, c) MODGUL & MODHEW: Aguilar-Llanos *et al.*, (2023). ACS Omega, 8, 42632-42646.

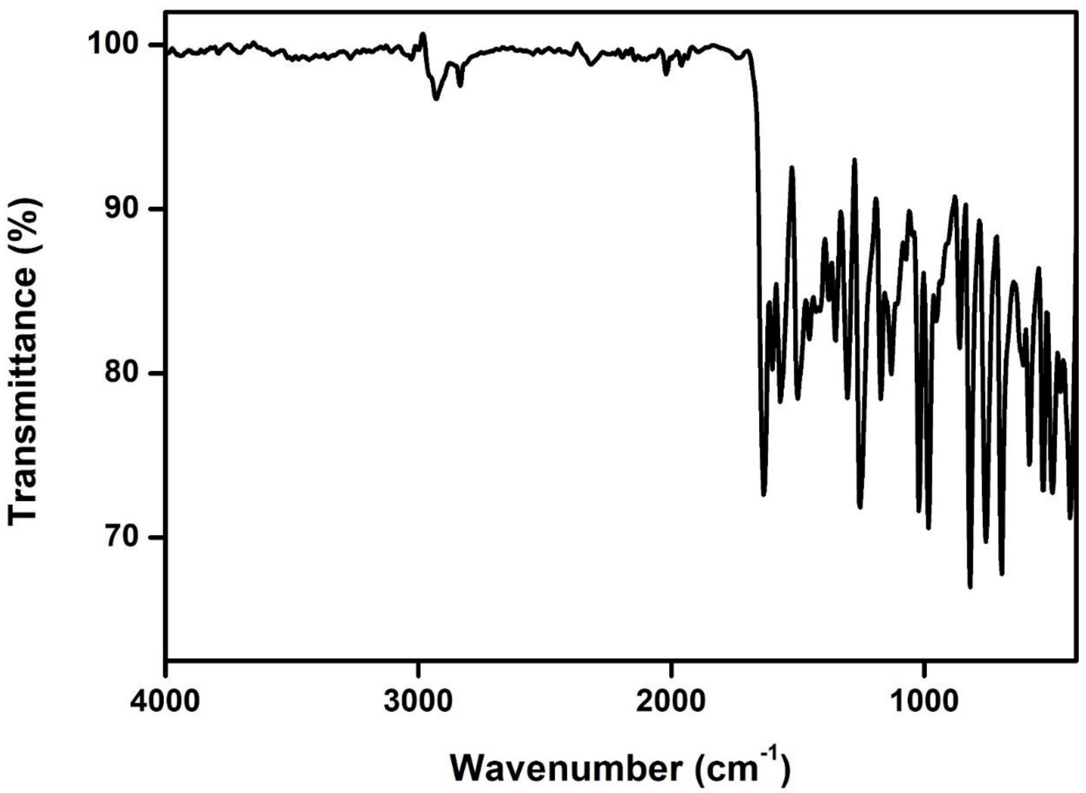


**Figure S1. FTIR spectrum of I**

***Fourier Transform Infrared (FTIR)***

Fourier Transform Infrared (FTIR) spectrum was recorded using an Agilent Cary 630 FTIR bench-top spectrometer in the range 400–4000 $cm^{-1}$ employing the KBr pellet technique. FTIR spectrum of compound **I** is shown in Fig. S1. A strong absorption band at 1602 $cm^{-1}$ is assigned to the azomethine (C=N) stretching vibration, confirming the successful condensation between the 4-aminoantipyrine and 4-methoxycinnamaldehyde. The band observed at 1635 $cm^{-1}$ is attributed to the carbonyl (C=O) stretching vibration of the antipyrine ring, while the absorption at 1570 $cm^{-1}$ corresponds to the aromatic C=C stretching vibrations. The strong band observed at 1022 $cm^{-1}$ is characteristic of the C-O stretching modes of the $CH_3O$ group of the 4-methoxycinnamaldehyde moiety.

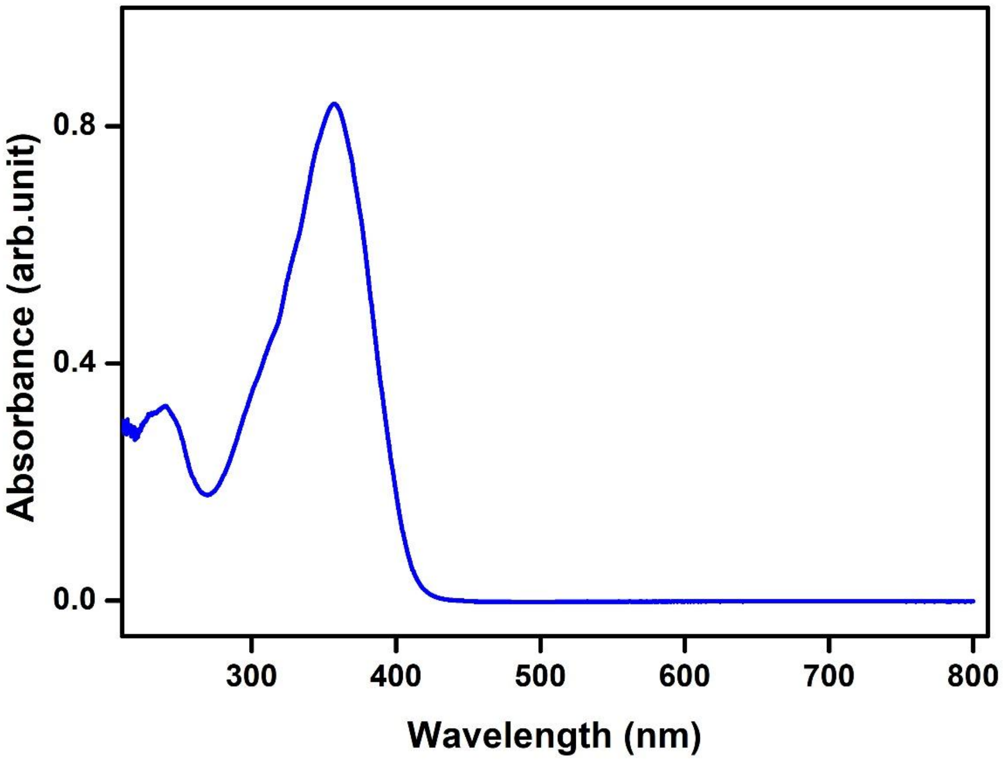


**Figure S2. UV-Vis spectrum of I**

### *UV-Vis absorption spectrum*

The UV-Vis absorption spectrum of **I** in ethanol was recorded using a JASCO 750-UV-Vis spectrophotometer in the range 200–800 nm. The electronic absorption spectrum of the compound exhibits two prominent absorption bands in the ultraviolet–visible region (Fig. S2). These can be attributed to characteristic electronic transitions within the molecular framework. The intense absorption band observed at 359 nm is assigned to the $\pi \rightarrow \pi^*$ transition arising from the aromatic conjugated system, predominantly involving transitions from the π-bonding orbitals of the phenyl and azomethine-linked pyrazole rings to their corresponding π*-antibonding orbitals. This red-shifted absorption indicates extended π-conjugation within the Schiff base framework. The second absorption band appearing at 240 nm is attributed to an $n \rightarrow \pi^*$ transition, originating from the non-bonding electrons of the carbonyl oxygen atom of the antipyrine moiety to the π* antibonding orbitals associated with the azomethine (–C=N–) linkage.

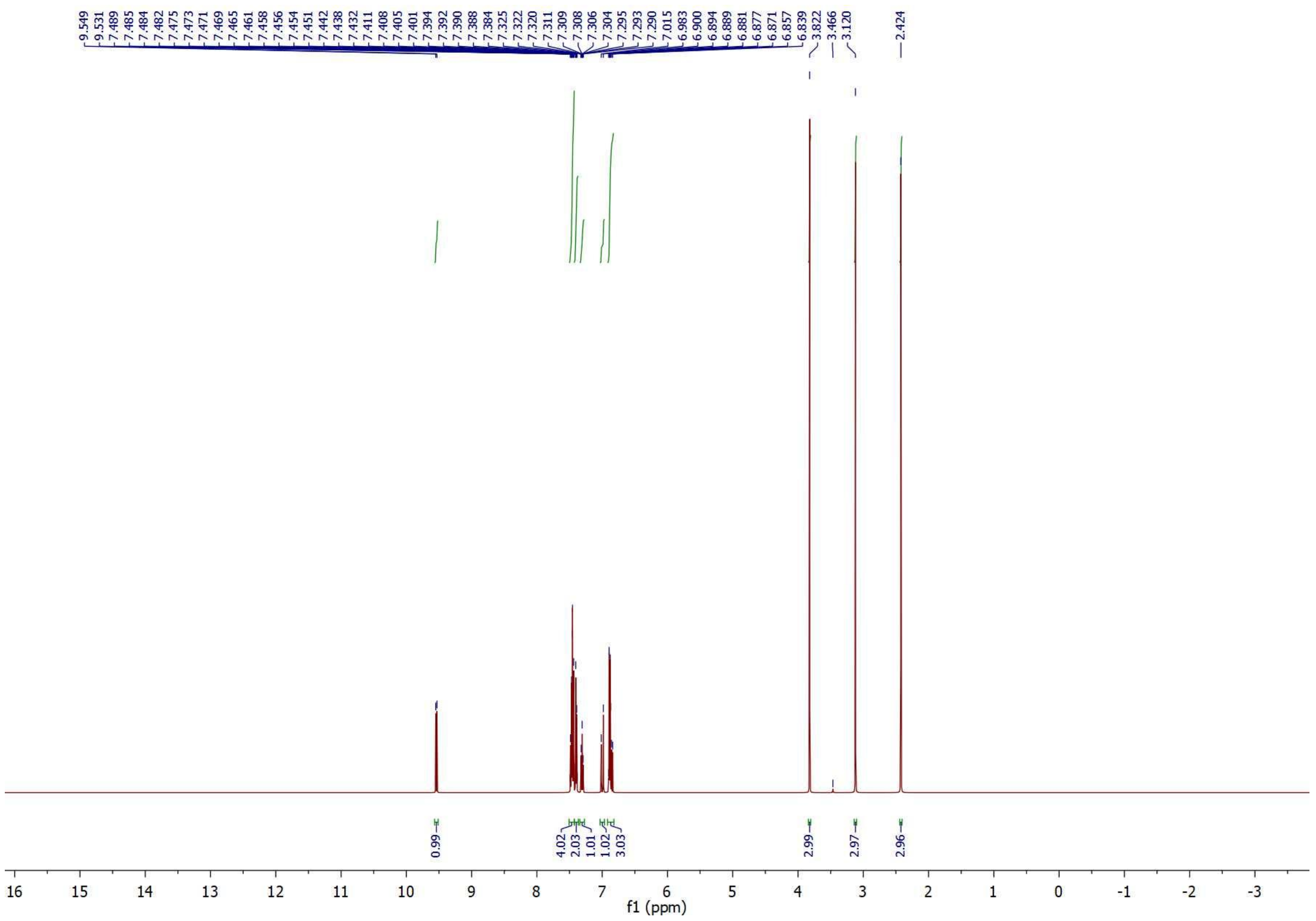


**Figure S3. $^1$H NMR spectrum of I**

### *$^1$H NMR spectrum*

$^1$H NMR spectrum of compound **I** (Fig. S3) was recorded on a Bruker 500 MHz spectrometer in $CDCl_3$ using TMS as the internal standard. In the $^1$H NMR spectrum, the downfield singlet at δ = 9.531–9.549 ppm is assigned to the imine (-CH=N-) proton, while the aromatic protons of 4-aminoantipyrine moiety resonate as multiplets in the regions δ = 7.489–7.432 ppm and 7.325–7.290 ppm. Aromatic protons of 4-methoxycinnamaldehyde moiety appear at δ = 7.411–7.384 ppm and 6.900–6.839 ppm. The olefinic protons of the 4-methoxycinnamaldehyde moiety are observed in the region δ = 7.015–6.983 ppm, partially overlapping with the aromatic signals at δ = 6.900–6.839 ppm. Further structural confirmation is provided by the aliphatic region, which exhibits a singlet at δ = 3.822 ppm corresponding to the methoxy group of the 4-methoxycinnamaldehyde moiety, along with two singlets at δ = 3.120 and 2.424 ppm attributable to the N-methyl and C-methyl groups of the aminoantipyrine moiety, respectively.

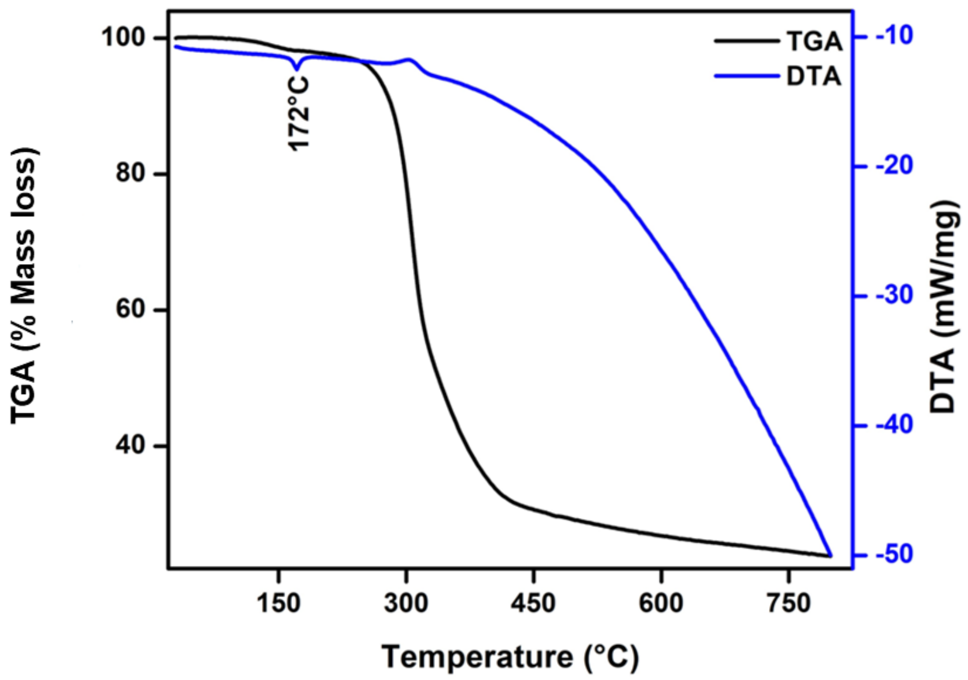


**Figure S4. Thermogravimetric analysis (TGA-black) and differential thermal analysis (DTA-blue) of I.**

***TGA/DTA***

The thermal properties of compound (**I**) are shown in Fig. S4. They were measured by thermogravimetric analysis (TGA) and differential thermal analysis (DTA) using a NETZSCH STA 2500 simultaneous thermal analyser over the temperature range of 30 to 800 °C, at a constant heating rate of 10 K $min^{-1}$. A sample of 2.491 mg was used for the measurement. The DTA curve indicates that the melting point of (**I**) is 172 °C/445 K. The TGA curve indicates that compound (**I**) is stable to *ca.* 300 °C/573 K after which a single-stage weight loss is observed.

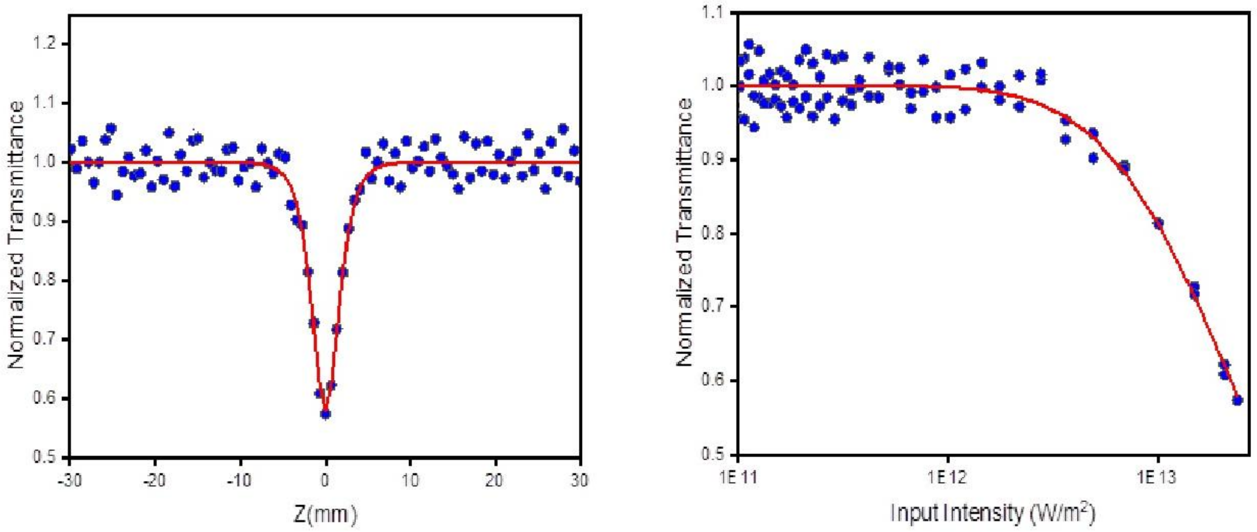


**(a)** **(b)**

**Figure S5.** **(a) Z scan curve for I, (b) Energy transmission *vs*. input fluence for I**

***Optical limiting behaviour***

The nonlinear absorption coefficient (β) of the title compound was measured using the single-beam Z-scan open aperture technique (Sheik-Bahae et al., 1990), with an Nd:YAG laser at 532 nm, a 10 Hz repetition rate, and a 9 ns pulse duration. The laser pulses, each with 100 μJ energy, were focused through a 15 cm focal length lens, creating a beam radius of 16.9 μm at the focus. A sample was dissolved in DMF and measured using a 1mm path-length quartz cuvette containing the dissolved sample. It was confirmed that the pure solvent showed no significant absorption under these conditions. Detailed methods for calculating the nonlinear absorption coefficient and optical limiting behaviour are available elsewhere (Subashini et al., 2013). The Z-scan curves (Fig. S5a) show smooth, symmetric valleys near the focus, indicating stronger absorption at higher intensities. The nonlinear absorption coefficient (β) of the compound is $5.20 \times 10^{-11}$ m/W. The optical limiting behaviour, shown as energy transmission *versus* input fluence (Fig. S5b), confirms that compound I has strong optical power limiting properties, with a limiting value of $4.93 \times 10^{12}$ W/m$^2$.

References


Sheik-Bahae, M., Said, A. A., Wei, T., Hagan, D. J. & Van Stryland, E. W. (1990). IEEE J. Quant. Electron, 26, 760-769.

Subashini, A., Leela, S., Ramamurthi, K., ArakCheeva, A., Stoeckli-Evans, H., Petricek, V., Chapuis, G., Pattison, P. & Philip, R. (2013). CrystEngComm, 15, 2474-2481.
